# Preconditions for successful guideline implementation: perceptions of oncology nurses

**DOI:** 10.1186/1472-6955-10-23

**Published:** 2011-11-08

**Authors:** Kaori Yagasaki, Hiroko Komatsu

**Affiliations:** 1 Faculty of Nursing and Medical Care, Keio University, 35 Shinanomachi, Shinjuku-Ku, Tokyo, Japan

## Abstract

**Background:**

Although evidence-based guidelines are important for improving the quality of patient care, implementation in practice is below expectations. With the recent focus on team care, guidelines are intended to promote the integration of care across multiple disciplines. We conducted an exploratory study to understand oncology nurses' perceptions of guideline implementation and to learn their views on how their experiences affected the implementation.

**Methods:**

A qualitative study was used with focus group interviews. We collected data from 11 nurses with more than 5 years of oncology nursing experience in Japan. The data were analyzed using grounded theory.

**Results:**

Results of the analysis identified "preconditions for successful guideline implementation" as a core category. There were 4 categories (goal congruence, equal partnership, professional self-development and user-friendliness) and 11 subcategories related to organizational, multidisciplinary, individual, and guideline levels.

**Conclusions:**

Although the guidelines were viewed as important, they were not fully implemented in practice. There are preconditions at the organizational, multidisciplinary, individual, and guideline levels that must be met if an organization is to successfully implement the guideline in clinical settings. Prioritizing strategies by focusing on these preconditions will help to facilitate successful guideline implementation.

## Background

The search for evidence has been an integral part of nursing since Florence Nightingale [1]. With the growing momentum of evidence-based practices, nursing specialty organizations have been active in developing practice guidelines [2-4]. Such guidelines are effective evidence-based tools to assist users in making appropriate clinical decisions and improving patient care [5-7]. The government agencies also encourage organizations to use the guidelines to reduce the variation in healthcare services and to promote standardization across the healthcare sector [8].

Another important aspect of meeting the diverse needs of cancer patients and their families is the team approach to multidisciplinary care [9,10]. The term "multidisciplinary" refers to a group of different disciplines, but the team concept is integral to the functioning of multidisciplinary care [11]. Xyrichis performed a concept analysis of teamwork, and suggested that the attributes of teamwork included concerted effort, interdependent collaboration and shared decision-making [12]. Multidisciplinary care is defined as "an integrated team approach to health care in which medical and allied health care professionals consider all relevant treatment options and develop collaboratively an individual treatment plan for each patient" [13].

Since practice guidelines promote consistency of knowledge and practice among team members [14] and improve the processes and outcomes of care [15], the guideline is helpful for oncology multidisciplinary care providers who are expected to practice evidence-based medicine [9]. However, as the previous study shows that differences among practitioners relate to the endorsement of and intention to use cancer guidelines [16], multidisciplinary care further complicates implementation.

An increasing number of Japanese professional societies have developed their own practice guidelines in addition to the Japanese versions of the American Society of Clinical Oncology (ASCO) and the National Comprehensive Cancer Network (NCCN) guidelines. In nursing, international guidelines such as the Best Practice Guideline of the Registered Nurses' Association of Ontario (RNAO) and Putting Evidence Into Practice: Improving Oncology Patient Outcomes (PEP) of Oncology Nursing Society (ONS) have been or being translated into Japanese. Although it is easy to access these guidelines on the Internet or in publications except the PEP which will be published soon, they are not systematically used by the health care organizations.

Not only in Japan bust also globally, the utilization of such guidelines in practice is below expectations [5,7]. There is a "gap between what we know works and what is actually done," and a large number of patients do not receive the recommended care [17-19]. The implementation of guideline can be complex. Previous studies have suggested that multidimensional factors, including patients, healthcare providers, organizations, and the environment, are intertwined with regard to the use of the guidelines [5,17,20]. A systematic review from Grimshaw et al. concluded that there was an imperfect evidence base to support decisions about which guideline dissemination and implementation are likely to be efficient under different circumstances [21].

To understand the complex phenomena involved in the process of guideline implementation, we need a qualitative study. Our focus in the present study was on the perceptions of oncology nurses regarding guideline implementation, as oncology nurses are well positioned to disseminate best practice information, and thus their views are critical for successful guideline implementation [22,23].We conducted an exploratory study to understand oncology nurses' perceptions of guideline implementation and to learn their views on how their experiences affected the implementation.

## Methods

### Design

Our focus was on oncology nurses' perceptions of guideline implementation within the realities of everyday clinical practice. Symbolic interactionism theory assumes that people create meaning through social interaction [24]. Grounded theory is the inductive discovery of a theory by analysing data in terms of a particular phenomenon [25]. We used the grounded theory approach based on symbolic interactionism to collect data about the behaviors of oncology nurses through focus interviews, and to identify their interactions with the guidelines, with others, and with the environment, with the goal of discovering how these interactions influence the implementation of guidelines.

### Participants

We conducted an open sampling in this study, and at a chemotherapy workshop we purposefully selected 30 oncology expert nurses who had basic knowledge of oncology care guidelines to participate in our study. Each nurse had more than 5 years of oncology nursing experience and had attended a training session on the use of guidelines in Japan. The nurses studied chemotherapy for 6 months and held oncology nursing certifications (certified nurse), issued by the Japanese Nursing Association, and differed from master-level clinical nurse specialists. Of the 30 selected oncology nurses, 11 agreed to participate in the study, while 19 elected not to participate, possibly because the focus group interview day was not convenient for them or they lived outside of Tokyo.

The study sample included expert nurses with a mean experience of 12 years (range, 9-17 years; SD, 2.53) from university-affiliated, general or cancer hospitals (range, 200-800 beds) and different locations in Japan (Table 1).

**Table 1:** Sample Characteristics (n = 11)

Demographics	
Female, n (%)	11 (100)
Age, mean (s.d.)	33.3 (2.9)
Specialty	
Years of Experience in Nursing, mean (s.d.)	12 (2.5)
Certified Nurses, n (%)	11 (100)
Type of Institution, n (%)	
General Hospital	6 (54.5)
Cancer Center	2 (18.2)
Designated Cancer Care Hospital	2 (18.2)
University-affiliated Hospital	1 (9.1)

### Data Collection

We conducted two focus group interviews at a nursing school in Tokyo in March 2009. We collected data from focus group interviews, because group dynamics would generate rich information and creative ideas. An investigator took on the role of facilitator and conducted two focus groups on different days using a semi-structured interview guide with open-ended questions (Table 2). The interview guide was developed by the authors based on previous studies [5,7,17,20]. The participants were informed of the purpose of the study and the discussion themes. The duration of the focus groups ranged from 60 to 80 min. The group interview was recorded by a digital sound recorder and transcribed verbatim, and the facilitator took notes during the session with the permission of the participants. The data reached saturation when no further unique theme emerged.

**Table 2:** Focus Group Interview Guide

**Focus groups were designed to answer the following questions regarding implementation of the guidelines**.
1. Adaptation and implementation of guidelines in clinical practice
1) Do you use the guidelines in practice?
2) Factors affecting the use of the guidelines • By whom, how, and how frequently are the guidelines used? • What are the challenges for implementing the guidelines?
3) Factors affecting non-use of the guidelines • Why is it difficult to use the guidelines in practice? • What factors affect the non-use of the guidelines?
4) What are the barriers for implementing the guidelines?
2. Regarding the guidelines
1) Did you find the contents that you wanted or expected in the guidelines?
2) What aspect regarding the use of the guidelines was difficult to understand?
3) What advantages and disadvantages did you find in adapting the guidelines in practice?
3. Strategies for implementing guidelines in practice
1) What strategies do you think were effective for applying the guidelines to individual patients?
2) What challenges and solutions do you think are effective for implementing the guidelines in practice?

### Data Analysis

The data were analyzed using the grounded theory approach [25]. All transcripts were reread, and the data were examined line by line (including a word-by-word microanalysis) for initial coding. The information was categorized by open coding (that is, conceptualizing on the first level of abstraction), and axial coding was subsequently performed to make conceptual connections between a category and its subcategories to identify the investigated phenomena. The category and sub-categories were further refined by selective coding to identify the core category.

To ensure the validity of this study, a single investigator performed all analyses, and this investigator discussed the interpretation and saturation of the data with a second investigator, who was an experienced qualitative researcher in nursing. In addition, a nurse sociologist verified the data and categorization.

### Ethical Considerations

All participants were informed of the voluntary nature of the study and their right to decline to participate. Written informed consent for study participation was obtained accordingly. The study was approved by the Ethics Committee of St. Luke's College of Nursing prior to initiating the study.

## Results

**Figure 1:**
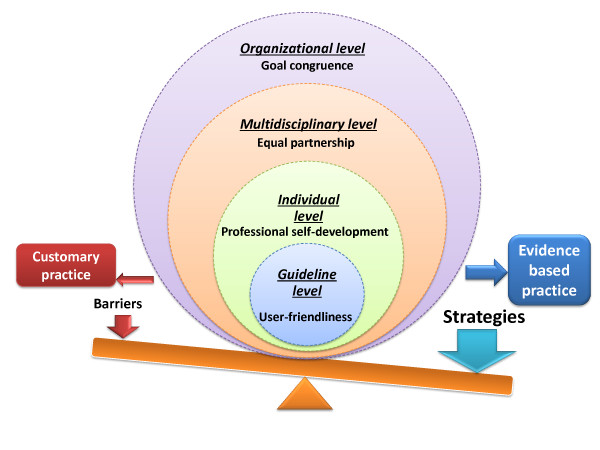
**Preconditions for Successful Guideline Implementation**.

All 11 oncology nurses participated in education for evidence-based nursing practice and had tried to implement guidelines on cancer treatment and chemotherapy in practice. Although there was a consensus among the participants that the implementation of guidelines is important, this study revealed that guideline implementation is still in the early stage. The analysis revealed "preconditions for successful guideline implementation" as a core category related to four categories at four levels (Figure 1). The study also identified 11 subcategories, 68 codes, and 293 labels (Table 3).

**Table 3:** Common themes in oncology nurses' descriptions of guideline implementation

Core category: Preconditions for successful guideline implementation	
Categories	Subcategories
Organizational level Goal congruence	• Resistance to change
	• Brand-loyal organization
	• Weak collaborative network with researchers
	• Lack of disseminators
	• Cost-conscious organization
Multidisciplinary level Equal partnership	• Poor consensus building across multiple disciplines
	• Obedience to physicians and senior staff
Individual level Professional Self-development	• Lack of guideline knowledge, judgment and skills
	• Detachment from the guideline
	• Workload pressure
Guideline level User-friendliness	• Impractical for routine use

### "Preconditions for Successful Guideline Implementation" as a Core Category

There are "preconditions for successful guideline implementation," which was revealed to be a core category characterized by four categories at four levels (organizational, multidisciplinary, individual, and guideline).

First, goal congruence is critical at the organizational level. Guideline implementation was hindered mostly by organizational factors, including resistance to change, brand-loyal organization, weak collaborative network with researchers, lack of disseminators, and cost-conscious organization. The oncology nurses perceived various challenges such as poor consensus building across multiple disciplines and the team dynamics in relation to obedience to physicians and senior staff at the multidisciplinary level, calling for equal partnership. At the individual level, professional self-development is essential. The nurses felt implementation was hampered by a lack of guideline knowledge, judgment and skills, detachment from the guidelines, and workload pressure. User-friendly guidelines are needed, because the oncology nurses expressed that the guidelines were impractical for routine use due to their overwhelming volume and the difficulties inherent in understanding the recommendations. These factors influence guideline use in the clinical setting, and they work as preconditions for successful guideline implementation.

#### 1. Organizational Level: Goal congruence

Goal congruence means that organizational goals are shared by all with support of the associated operations and activities. This category emerged as an important precondition to achieve the goal of implementing the guideline at the organizational level. The nurses perceived that the value of guideline implementation was not shared throughout the organization, although shared goals would facilitate constructive discussion.

##### Resistance to Change

When motivated nurses tried to introduce a new procedure based on the guidelines, they faced the emotional resistance of team leader to change and the resistance of team members in daily practice.

*Even though I presented evidence, the physician showed his emotional resistance to the guidelines, as he has never understood them (Participant D)*.

*I presented evidence, but the physician and hospital management said there has been no problem so far (Participant C)*.

*Regarding changing of needles, a physician asked why nurses insist on changing them because it's no problem (to re-use a used one). We had such an argument (Participant G)*.

On the other hand, the nurses anticipated constructive discussion and shared-values among multidisciplinary members when following the guidelines:

*The guidelines have authority; if a certain instruction is described in the guidelines, nobody will completely refuse it in front of their co-workers. Also, the guidelines give us a good opportunity for discussion (Participant I)*.

##### Brand-loyal Organization

The nurses indicated that organizations were inclined to "follow a name value rather than the guidelines". Organizational values and culture impede the utilization of guidelines.

*Even though something is recommended in the guidelines, somebody would say that a certain hospital is doing it like this. In such a case, "me-tooism" occurs, and it is difficult to breakaway from the consensus (Participant F)*.

*It is hard to change one's attitude (value) when following a brand (Participant I)*.

##### Weak Collaborative Network with Researchers

The oncology nurses faced the resistance of organizations, supervisors, and other professionals, and felt that "there are limitations if we try to disseminate information or make changes by ourselves". The participants addressed the need for collaboration with researchers.

*It doesn't work for me, even if I work hard. I still need the help of other people and various networks (Participant G)*.

*We are not able to determine whether the guidelines constitute real evidence, so we need to work together with researchers as they are the ones who acquire the evidence, and then we want to use the guideline in practice (Participant E)*.

*We also need companions to work with. We hope that the researcher determines whether or not appropriate evidence exists. We want to present the necessary information together with the researchers (Participant F)*.

##### Lack of Disseminators

The participants who were not comfortable using the guidelines in practice expected a leader to develop a network to disseminate information. They perceived the lack of guideline disseminators as a hindrance. The chemotherapy relevant committees were established in individual institutions, but there were no professionals who have knowledge of manual development and procedures relevant to practice. The participants demanded disseminators to promote the guidelines for appropriate use.

*We need to inform others of the availability of the guideline...somebody needs to promote it (Participant J)*.

*We need a person who can clearly tell why we need to have evidence (Participant I)*.

The oncology nurses noted *"we need to foster professionals who disseminate new ideas in our institution" (Participant J)*, suggesting the development of human resources and the establishment of a worthy role model for other staff to promote the guidelines.

##### Cost-conscious Organization

The participants recognized that *"costs will be a barrier when we make a change". "The organization tries to reduce costs when they are high even though evidence in the guidelines recommends otherwise" (Participant K)*. Relevant to this, *"even though the evidence is clear, we cannot use it due to the policies of a certain physician or hospital management"*, suggesting *"the organization lacks interest in evidence-based practices" (Participant I)*.

*As for gloves (for chemotherapy), we presented the guidelines, but because additional cost would have been incurred, the administrator resisted spending by saying "Well...". On the other hand, if something can be reimbursed, the administrators change their attitudes (positively) (Participant H)*.

*I think the organization should recognize the meaning and value of the guidelines. There seems to be a fundamental consensus that evidence-based guidelines are important. Without organizational support, both physicians and nurses follow superficial procedures (Participant K)*.

The participants also noted that financial reimbursement for work specified by the guidelines would be an important driving force for the organization.

*Since financial matters are important for management, administrators will implement the guidelines if costs are reimbursed (Participant I)*.

*If for example, extra chemotherapy work specified by the guidelines is reimbursed, we will get serious about the guidelines (as an organization) (Participant H)*.

#### 2. Multidisciplinary Level: Equal partnership

Equal partnership emerged as a precondition at the multidisciplinary level in which all professionals would have equal rights to participate in decision-making.

##### Poor Consensus Building across Multiple Disciplines

The oncology nurses addressed the lack of communication and information sharing and the difficulty of reaching a consensus regarding the use of the guidelines across multiple disciplines. For example, extravasation care of chemotherapy drugs and changes of procedures are practiced by not only nurses but also other professionals, including physicians, oncologists, dermatologists, and pharmacists. The interviews revealed that there was no formal system for routine discussion of such matters among multidisciplinary members. Furthermore, there was no established consensus-building process among multidisciplinary members in daily practice.

*Since nurses are not in the position to effect change, we need physicians' involvement...but we don't know how to get physicians involved (Participant H)*.

The oncology nurses required opportunities to share their goals and discuss decision making among multidisciplinary members. In addition, they believe that the more professionals discuss the guidelines in daily practice, the more utilization of the guidelines will be promoted.

*If there is a multidisciplinary committee where all the members can share problems, then, we believe we can do it (Participant B)*.

The participants perceived that these challenges were not limited to nurses, but the knowledge, skills, and judgment of physicians and pharmacists should be improved as well.

*We feel that instruction on how to use the guidelines needs to be taught to not only nurses but also physicians (Participant J)*.

##### Obedience to Physicians and Senior Staff

A sense of obedience to physicians in hospitals, particularly in rural areas, is deeply rooted in clinical practice insofar that healthcare providers "*strongly believe that what the physician says is absolutely right*". In addition, *"it is very difficult to say 'No' to conservative senior staff" (Participant A)*.

*In our hospital, what the physician says is absolutely right; for example, the regimen the physician orders are always right (Participant K)*.

*(To improve these situations,) *"*First of all, nurses should be aware of evidence (guidelines). Educational programs are necessary for nurses" (Participant B)*.

#### 3. Individual Level: Professional self-development

Individual nurses perceived that they needed professional self-development for knowledge and skills to implement the guideline. Professional self-development is a key precondition at the individual level.

##### Lack of Guideline Knowledge, Judgment, and Skills

Although the oncology nurses attempted to use the guidelines in practice, they recognized their limited abilities when searching for resources and making decisions about applying the guidelines to individual patients.

*We can't use the guidelines unless we can access them, but we don't know how to access particular resources (Participant A)*.

*We are not able to determine whether we could apply the guidelines for individual patients (Participant E)*.

The nurses were aware of the need to improve their skills through educational programs in oncology nursing.

*We definitely need to acquire the skills for accessing new information and employing it in a clinical setting (Participant E)*.

##### Detachment from the guidelines

Implementation of the guidelines was hindered by "not-my-business" attitudes and lack of interest in guidelines and passive and non-independent attitudes.

*We were not familiar with the guidelines. We felt it was not our business*.

Even though the guidelines are accessible to the unit, maybe we will not see

*them (Participant F)*.

The participants addressed the need of a working group because *"first of all, it is necessary to raise awareness of guidelines in daily practice"*. Some participants noted that their role and responsibility could lead to behavioral changes among healthcare providers.

*When asked about new chemotherapy knowledge in the guidelines, it reminded me of my role as an oncology nurse (Participant D)*.

##### Workload Pressure

The nurses raised concerns over workload pressures in their already over-burdened profession, as they have no time to find new information during busy working time. Furthermore, they had to overcome a complicated organizational system to change routine practices by using the guidelines; therefore, they deferred dealing with guidelines.

*I can't find time to consult the guidelines after hours (Participant J)*.

*Even if I want to check something, I don't have the time to look it up immediately, so I leave it on the back burner (Participant F)*.

The nurses demanded easy access to guideline information:

*If something is easy to find and apparent just by looking, and can be used immediately (Participant I)*.

*I want to access the guideline via the Internet...but I don't know where and what kinds of guidelines are available. If I did know, I would use it, but such information does not seem to be at my fingertips (Participant A)*.

#### 4. Guideline Level: User-friendliness

User-friendliness is important for both guideline developers and users. It is a precondition at the guideline level.

##### Impractical for Routine Use

The guidelines themselves were viewed as a barrier because they were not presented in an accessible and user-friendly format. The nurses perceived that the guidelines were impractical for routine use because of lack of access and the difficulty in understanding recommendations in the guidelines.

*The guidelines often say that something is very difficult (Participant A)*.

*It is not easy to find the information we want (Participant B)*.

*I am too busy to read "all the recommendations" (Participant A)*.

*It is impractical unless it is handy (Participant H)*.

The nurses addressed the difficulty and complexity of using the guidelines in a clinical setting and stated that they should be modified for clinical practice.

*I believe it is necessary to modify the guidelines to ensure feasibility in a clinical setting and that even a novice nurse can use them (Participant G)*.

The interviews revealed that the implementation of the guidelines was affected by factors at the organizational, multidisciplinary, individual, and guideline levels. The development of the guidelines was not directly linked to their immediate use in practice due to various factors that were interlinked in complex ways.

## Discussion

This study highlights preconditions for successful guideline implementation based on oncology nurses' perceptions. Many factors influence the implementation of the guidelines at multiple levels. Therefore, future implementation strategies will be focused on a specific precondition at each level: goal congruence at the organizational level, equal partnership at the multidisciplinary level, professional self-development at the individual level, and user-friendliness at the guideline level.

There were some differences between the results of our studies and the literature. While Gurses et al. reported physician's disbelief of guidelines as a barrier to guideline implementation in their review, lack of agreement with a guideline or skepticism was not found in nurse participants in our study [26]. Also, any patient factor, which might influence guideline implementation according to the systematic review [15], was not identified as a precondition in our results.

In this study, goal congruence at the organizational level was revealed as an important precondition to implement guidelines. Priorities are often different between the organization and front line staff. Goal congruence is the first vital step to implementing the guidelines. The guidelines were supposed to represent a strategic change towards best practice [27]; however, organizational factors often negatively influence guideline implementation. In this study, there were five challenges at the organizational level, which had the largest number of challenges among the four levels.

With ambiguous rationale, health care providers resist changes or easily become captivated by brand names. Funk et al. addressed the responsibility of researchers for scientific aspects, administrators for the institutional environment for research use, and practitioners for guideline implementation [28]. In addition, disseminators of the guidelines must facilitate the change from customary practice to evidence-based practice.

Since organizations are cost-conscious, costs create obstacles for adopting the guidelines. In reality, organizations need financial incentives to change established routines. As the interviewed oncology nurses argued, reimbursement could be a strong incentive for successful guideline implementation [7]. At the other end of the spectrum, guideline implementation should be cost-effective in principle [15]. Proving the cost effectiveness within the framework of the insurance system would be a driving force for change, and thus it is urgent to explore such an agenda.

Working in equal partnership among multidisciplinary team members is imperative for guideline implementation at the multidisciplinary level. The hierarchy among healthcare providers impedes the use of the guidelines. Nurses as well as other staff are likely to depend on physicians or senior staff. Individual healthcare providers should be independent as professionals.

Cancer care requires a multidisciplinary team approach. With multiple disciplines involved, sharing information towards a common goal beyond the boundaries of the profession is essential, and the guidelines can be an efficient tool. Although the guidelines should be incorporated into practice upon consensus among professionals [29], poor consensus building across multiple disciplines is a serious challenge for team care. One of the reasons behind this is that there are different norms in different disciplines, which reportedly affects team care [11]. Discipline-specific interests may hinder understanding and respect for the perspectives of members of other disciplines. Teamwork creates a work culture that values collaboration. Equal partnership is a prerequisite for true collaboration. The need for collaborative professional work is more important than ever in the era of patient-centered care.

Professional self-development is the key at the individual level. Nurses should prepare themselves to respond to changing needs in health care, and they are responsible for their own development. The results of this study suggest that professional development to update knowledge should be incorporated as a strategy for guideline implication. This study targeted only oncology nurses who had specialty education in chemotherapy, yet even these nurses with relatively high levels of education perceived their individual abilities as limited. They are not comfortable using the guidelines or are not able to make decisions about whether the guidelines are applicable to their own patients. Melnyk et al. reported that nurses' knowledge about evidence-based practice was much lower than their beliefs that evidence-based practice improves clinical care and patient outcomes [30].

Commitment is always required to introduce something new. Some nurses, however, feel detached from the guidelines, which represents their passive attitudes and limited interest. Negative staff attitudes and beliefs [5], lack of professional accountability [31], and lack of familiarity with the guidelines [22,32] have been identified as barriers in the literature. Oncology nurses should be aware of the impact of their knowledge, morale, and behavior as professionals, and of their responsibility to utilize the guidelines [26].

The oncology nurses expressed their concerns regarding the additional workload due to the adaptation of the guidelines. This hurdle could be overcome by motivation and professional commitment as a nurse as well as by support from a nursing manager and colleagues [5]. Interestingly, a previous study noted that the practitioners in the clinic where the guideline was not used mentioned lack of time as a barrier, while those in the clinic where the guideline was used did not report this problem, probably due to the attempt to practice change [32].

Lastly, further improvement of the guidelines is needed. The quality of clinical practice guidelines often varies widely [33]. Despite a recommended external review of the draft guidelines before dissemination as a part of the development phase [6], the current format of the guidelines is also noted as a concern because of their great length, their complicated nature, and lack of opportunity to test them [26,34]. These hard-to-use and complex features of the guidelines may discourage nurses and decrease their expectations [17]. It is feasible for guideline developers to modify the content and format of guidelines in consideration of implementability [35]. AGREE II is a useful tool for health care providers to appraise the guideline [36]. Indeed, when we developed the chemotherapy guideline, an external evaluation was performed using the Japanese version of AGREE [37], the volume of the guideline and applicability to the local context persuaded us to revise the guideline draft [38].

The oncology nurses voiced the need for a collaborative network with researchers who need to incorporate the views of practitioners into the development of the guidelines and support practitioners for dissemination, even after implementation. Such collaborative efforts could lead to sustainable guideline utilization.

This study has implications for policy and clinical practice. Japan has established the initiatives to improve the quality of cancer care, begun when the Cancer Control Act was approved in 2006. The use of guideline has been encouraged for standardization of treatment. Preconditions derived from practical issues at different levels identified in this study will provide policy makers with a better understanding of practitioners' perceptions, and help practitioners to implement guidelines. Organizations must understand the significance of guideline implementation and its positive consequences, and set up procedures and activities to support the goal. Furthermore, organizations should strengthen their collaborative networks with researchers to facilitate guideline implementation.

## Limitations

The results of this study may be biased toward oncology nurses, including nurse managers, who were more educated for performing chemotherapy and evidence-based practices than are general nurses. We did not interview those from other disciplines in the multidisciplinary team, and therefore, generalizability to the wider inter-professions may be limited. However, the inclusion of hospitals offering different levels of care in not only urban but also rural area communities should increase the validity of the findings. We conducted only open sampling. We failed to conduct relational and variational sampling, and discriminate sampling. The limitations of the sampling and questions based on the semi-structured interview guide might influence the deeper exploration and generation of an emerging theory. Because of the nature of the focus group interview, the results may be influenced by group dynamics. The advantage of this approach is the acquisition of constructive data, while the disadvantage is the influence of the opinions of others. To maintain data validity, two investigators ascertained the analysis process, but there may have been limitations to this approach.

## Conclusions

This study elucidated the perceptions of oncology nurses regarding guideline implementation. Although the guidelines are seen as important, they are not fully used in practice. Development of the guidelines is not directly linked to their utilization, and there are preconditions at the organizational, multidisciplinary, individual, and guideline levels to successful implementation of the guidelines in clinical settings. Prioritizing strategies by focusing on these preconditions will help to facilitate successful guideline implementation. Future research is needed to assess the quality of care and cost effectiveness that can result from implementing the guidelines, and to develop professional self-development programs for clinical nurses.

## Competing interests

The authors declare that they have no competing interests.

## Authors' contributions

KY was responsible for the study conception and design, data analysis, and manuscript drafting. HK was responsible for the study conception and design, and critical revisions of the manuscript. All authors read and approved the final manuscript.

## Pre-publication history

The pre-publication history for this paper can be accessed here:

http://www.biomedcentral.com/1472-6955/10/23/prepub
